# Survival of patients with squamous cell carcinoma of the breast compared with invasive ductal carcinoma by biological subtype: A matched analysis of the Japanese national clinical database-breast cancer registry

**DOI:** 10.1016/j.breast.2025.104567

**Published:** 2025-08-27

**Authors:** Mami Ogita, Hiraku Kumamaru, Makoto Kubo, Naoko Kinukawa, Naoki Niikura, Shigehira Saji, Masakazu Toi

**Affiliations:** a Department of Radiology, The University of Tokyo Hospital, 7-3-1 Hongo, Bunkyo-ku, Tokyo 113-8655, Japan; b Department of Healthcare Quality Assessment, Graduate School of Medicine, The University of Tokyo, 7-3-1, Hongo, Bunkyo-ku, Tokyo, 113-0033, Japan; c Committee of Breast Cancer Registry and Data Science, 3-8-16, Nihonbashi, Chuo-ku, Tokyo, 103-0027, Japan; d Department of Breast Surgical Oncology, Kyushu University Hospital, 3-1-1, Maidashi, Higashi-ku, Fukuoka, 812-8582, Japan; e Department of Breast Oncology, Tokai University School of Medicine, 143, Shimokasaugaya, Isehara, Kanagawa, 259-1193, Japan; f Department of Medical Oncology, Fukushima Medical University, 1, Hikarigaoka, Fukushima, 960-1295, Japan; g Tokyo Metropolitan Cancer and Infectious Disease Center, Komagome Hospital, 3-18-2, Honkomagome, Bunkyo-ku, Tokyo, 113–8677, Japan

**Keywords:** Breast cancer, Squamous cell carcinoma, Metaplastic carcinoma, Radiation therapy, Prognosis, Recurrence, Survival, Registries

## Abstract

**Purpose:**

Owing to the rarity of primary squamous cell carcinoma (SCC) of the breast, the prognosis of SCC remains uncertain. We aimed to investigate the clinical features and prognosis of breast SCC by subtype.

**Methods:**

A total of 350,977 patients with breast SCC or invasive ductal carcinoma (IDC) were identified from the National Clinical Database-Breast Cancer Registry from 2004 to 2014. SCC and IDC patients with triple-negative and luminal subtypes were matched 1:1 via exact matching. Overall survival (OS), breast cancer-specific survival (BCSS), and recurrence-free survival (RFS) were compared between patients with SCC and those with IDC. In-field area recurrence was analyzed among patients who received adjuvant radiotherapy.

**Results:**

The study included 452 SCC patients and 182,707 IDC patients. SCC patients were more likely than IDC patients to have advanced-stage disease. The crude 10-year OS, BCSS, and RFS were 70 %, 80 %, and 66 % for patients with SCC, and 88 %, 93 %, and 81 % for patients with IDC, respectively. After 204 patients with the triple-negative subtype and 68 patients with the luminal subtype in each group were matched, the 10-year BCSS was significantly worse for SCC (76.7 %) than for IDC (85.5 %) within the triple-negative subtype. There were no differences in OS, BCSS, or RFS for the luminal subtype. The rates of in-field area recurrence were similar between patients with SCC and those with IDC with either the triple-negative subtype or the luminal subtype.

**Conclusions:**

Within the triple-negative subtype, SCC histology was associated with a significantly worse prognosis than IDC.

## Highlights

• SCC was diagnosed at an advanced stage and had a poorer prognosis than IDC.
• SCC was an independent worse prognostic factor for the triple-negative subtype.
• A sensitivity analysis using propensity score matching was performed.
• In-filed area recurrence appeared comparable between SCC and IDC.

## 1 Introduction

Primary squamous cell carcinoma (SCC) of the breast is rare, accounting for only 0.1 % of all breast cancers [1,2]. SCC is a form of metaplastic carcinoma originating from benign squamous metaplasia [3]. Owing to the rarity of SCC, most published studies on SCC are case reports or small retrospective cohorts [[4], [5], [6], [7], [8], [9], [10], [11]], and its prognosis and optimal management remain uncertain. Some studies suggest that SCC is more aggressive and associated with poorer prognosis than invasive ductal carcinoma (IDC) [[12], [13], [14], [15], [16], [17]].

SCC frequently presents a triple-negative phenotype, similar to other metaplastic cancers [3]. Triple-negative breast cancer (TNBC) shows rapid growth and a poor prognosis [18,19]. It is unclear whether the aggressive outcomes of SCC are driven primarily by its triple-negative status or by its histology. SCC is often treated like aggressive IDC using multimodal therapy—including radiotherapy—though the benefit of adjuvant radiotherapy remains unclear.

Therefore, we aimed to investigate the clinical characteristics of SCC and compare the prognosis and effectiveness of adjuvant radiotherapy between SCC and IDC by biological subtype.

## 2 Materials and methods

### 2.1 Data source and patients

We collected data from the National Clinical Database-Breast Cancer Registry (NCD-BCR) in Japan from 2004 to 2014. The BCR was established in 1975 by the Breast Cancer Research Society and became a web-based online registry in 2004. It merged with the NCD registry in 2012 and became the NCD-BCR. The NCD-BCR covers over 95 % of breast cancer cases in Japan and has obtained more than 900,000 patient records from over 1400 institutions. Initial prognosis follow-up data are recorded every five years from the initial treatment.

We included female breast cancer patients aged 18 years or older with either SCC or IDC histological types, invasive cancer, and stage I-III disease who underwent surgery. We excluded patients who received preoperative radiotherapy, had noninvasive cancer, had distant metastasis prior to treatment, or had histological types other than SCC and IDC. Patients without any follow-up data were also excluded. The diagnosis of SCC was based on the pathological criteria of each participating institution. No central pathology review was performed. The institutions contributing to this database are considered to meet a certain standard in pathological diagnosis.

### 2.2 Clinicopathological factors

Background information was obtained from the database. TNM classification and stage were based on the criteria by the American Joint Committee on Cancer. Hormone receptor (estrogen and progesterone receptor) status (ER and PgR) and human epidermal growth factor receptor 2 (HER2) status were determined from the pathological results of surgery. Preoperative pathological results were used when patients received neoadjuvant therapy or when surgical pathological results were unavailable. Hormone receptor expression was considered positive when at least 1 % of the nuclei of the tumor cells were stained. HER2 status was considered positive when HER2 expression was 3+ on the basis of immunohistochemistry (IHC) or 2+ on the basis of IHC along with positive fluorescence in situ hybridization. The biological subtypes were categorized as follows: luminal subtype (ER+ and/or PgR+ and HER2-), luminal–HER2 subtype (ER+ and/or PgR+ and HER2+), HER2 subtype (ER- and PgR- and HER2+), and triple-negative subtype (ER- and PgR- and HER2-).

### 2.3 Outcomes

We assessed overall survival (OS), breast cancer-specific survival (BCSS), recurrence-free survival (RFS), incidence of first locoregional recurrence, and first distant metastasis recurrence. OS, BCSS, and RFS were defined as the time from the first day of treatment to the date of any death, breast cancer death, or any recurrence or death, respectively. The incidences of first locoregional recurrence and first distant metastasis recurrence were defined as the time from the first day of treatment to the date of the first locoregional recurrence (with or without simultaneous distant metastasis) or the first distant metastasis recurrence (with or without simultaneous locoregional disease).

Among patients who received adjuvant radiotherapy, we examined in-field area recurrence. The data on locoregional recurrence sites were categorized according to the following sites: the ipsilateral breast, ipsilateral axillary lymph nodes, and ipsilateral local sites (skin, chest wall, or regional lymph nodes). In-field area recurrence was defined as recurrence within the irradiated area. Since radiation field data were not available, in-field area recurrence was used as a surrogate to estimate recurrence within the irradiated field.

### 2.4 Statistical analysis

The OS, BCSS, RFS, first locoregional recurrence, and first distant metastasis recurrence were estimated via the Kaplan‒Meier methods, and group differences were compared via the log-rank test. To adjust for potential confounding factors, an exact matching analysis was conducted. The matching variables included years of surgery, age, clinical T stage, clinical N stage, breast surgery, axillary surgery, chemotherapy, molecular targeted drug/antibody therapy, endocrine therapy, and radiotherapy. Continuous variables were converted into categorical variables for analysis. In exact matching, matched pairs are required to have identical values for all specified covariates, ensuring perfect balance without relying on modeling assumptions. A 1:1 matching ratio was applied. After matching, outcomes were compared between SCC patients and IDC patients within each of the two predominant SCC subtypes (the triple-negative and luminal subtypes). Covariate balance before and after matching was assessed to evaluate the effectiveness of the matching procedure.

We conducted a sensitivity analysis via 1:2 propensity score matching (PSM) without replacement with a caliper width set at 0.1 standard deviations of the logit of the propensity score. Covariate balance before and after PSM was assessed via standardized mean differences (SMDs). In the matched cohort, Cox proportional hazards models were used to estimate hazard ratios (HRs) and 95 % confidence intervals (CIs) for survival outcomes. The proportional hazards assumption was assessed to confirm the validity of the Cox models.

Cases with missing values for the matching variables were excluded. Similarly, PSM was performed using only cases with complete data for all covariates included in the model. Statistical significance was defined as a two-sided P < 0.05. All the statistical analyses were performed via SAS version 9.4 (SAS Institute, Cary, NC, USA).

## 3 Results

### 3.1 Patient population and characteristics

**Fig. 1:**
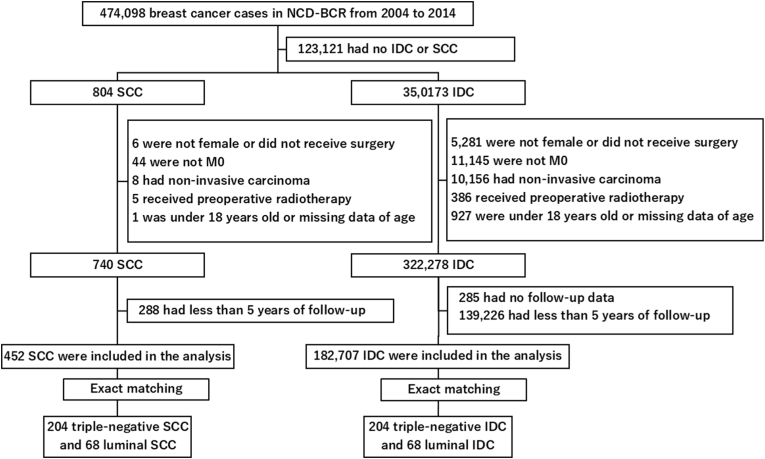
Flow diagram of participant selection. NCD-BCR: National Clinical Database-Breast Cancer Registry; SCC: squamous cell carcinoma; IDC: invasive ductal carcinoma.

Between 2004 and 2014, a total of 474,098 breast cancer patients were registered in the NCD-BCR. Among them, 804 patients had SCC, and 350,173 patients had IDC. The incidence of SCC among all breast cancers was 0.17 %. After ineligible patients were excluded, 452 patients with SCC and 182,707 patients with IDC were included in the analysis (Fig. 1). Table 1 shows the patient characteristics. Patients with SCC were more likely to have advanced-stage disease and undergo mastectomy, axillary lymph node dissection, and chemotherapy. The predominant biological subtype of SCC was triple-negative (58.8 %), followed by luminal (19.2 %), HER2 (8.0 %), and luminal–HER2 (2.0 %). Detailed information of chemotherapeutic agents and adjuvant radiotherapy is summarized in Supplementary Table 1. The most commonly administered chemotherapy regimen in both SCC and IDC was a combination of anthracyclines and taxanes. Adjuvant radiotherapy was performed in 65.7 % of SCC patients and 74.0 % of IDC patients after BCS, and in 17.8 % of SCC patients and 13.8 % of IDC patients after mastectomy (data not shown). The patient characteristics of each subtype before and after matching are shown in Supplementary Tables 2, 3, 4.

**Table 1:** Clinicopathological characteristics and treatment patterns for stage I-III SCC and IDC patients starting treatment in 2004–2014 in the NCD-BCR cohort.

		SCC		IDC	
		n = 452	%	n = 182,707	%
Age, years	−29	4	0.9	821	0.4
	30–39	22	4.9	11,262	6.2
	40–49	73	16.2	40,293	22.1
	50–59	123	27.2	43,200	23.6
	60–69	115	25.4	46,733	25.6
	70−	115	25.4	40,398	22.1
cT	T1	82	18.1	96,451	52.8
	T2	232	51.3	68,001	37.2
	T3	51	11.3	6528	3.6
	T4	82	18.1	8244	4.5
	Unknown	5	1.1	3483	1.9
cN	N0	282	62.4	142,590	78.0
	N1	130	28.8	33,124	18.1
	N2	27	6.0	4127	2.3
	N3	11	2.4	2334	1.3
	Unknown	2	0.4	532	0.3
cStage	I	70	15.5	87,899	48.1
	II	318	70.4	84,030	46.0
	III	57	12.6	6861	3.8
	Unknown	7	1.5	3917	2.1
Surgery	BCS	140	31.0	103,928	56.9
	Mastectomy	304	67.3	75,033	41.1
	No	6	0.4	2768	0.5
	Unknown	2	1.3	978	1.5
Axillary surgery	SLNB	156	34.5	90,026	49.3
	ALND	262	58.0	83,254	45.6
	No	30	6.6	9133	5.0
	Others	2	0.4	125	0.1
	Unknown	2	0.4	169	0.1
ER	Positive	98	21.7	142,909	78.2
	Negative	329	72.8	34,936	19.1
	Unknown	25	5.5	4862	2.7
PgR	Positive	40	8.8	120,748	66.1
	Negative	386	85.4	56,731	31.1
	Unknown	26	5.8	5228	2.9
HER2	Positive	45	10.0	28,047	15.4
	Negative	355	78.5	133,550	73.1
	Unknown	52	11.5	21,110	11.6
Subtype	Luminal-HER2	9	2.0	15,927	8.7
	Luminal	87	19.2	114,674	62.8
	HER2	36	8.0	11,997	6.6
	Triple-negative	266	58.8	18,302	10.0
	Unknown	54	11.9	21,807	11.9
Neoadjuvant therapy	Yes	113	25.0	30,388	16.6
	No	338	74.8	152,047	83.2
	Unknown	1	0.2	272	0.1
Neoadjuvant chemotherapy	Yes	109	24.1	25,053	13.7
	No	342	75.7	157,381	86.1
Neoadjuvant molecular targeted therapy	Yes	13	2.9	5655	3.1
	No	438	96.9	176,771	96.8
Neoadjuvant endocrine therapy	Yes	8	1.8	6403	3.5
	No	443	98.0	176,024	96.3
Adjuvant therapy	Yes	330	73.0	161,305	88.3
	No	117	25.9	19,032	10.4
	Unknown	5	1.1	2370	1.3
Adjuvant chemotherapy	Yes	266	58.8	58,328	31.9
	No	181	40.0	122,009	66.8
Adjuvant molecular targeted therapy	Yes	39	8.6	19,184	10.5
	No	408	90.3	161,147	88.2
Adjuvant endocrine therapy	Yes	69	15.3	128,666	70.4
	No	378	83.6	51,664	28.3
Adjuvant radiotherapy	Yes	148	32.7	88,840	48.6
	No	296	65.5	90,750	49.7
	Unknown	8	1.8	3117	1.7

SCC: squamous cell carcinoma; IDC: invasive ductal carcinoma; BCS: breast-conserving surgery; SLNB: sentinel lymph node biopsy; ALND: axillary lymph node dissection; ER: estrogen receptor; PgR: progesterone receptor; HER2: human epidermal growth factor receptor 2.

### 3.2 Comparison of crude clinical outcomes between SCC patients and IDC patients

**Fig. 2:**
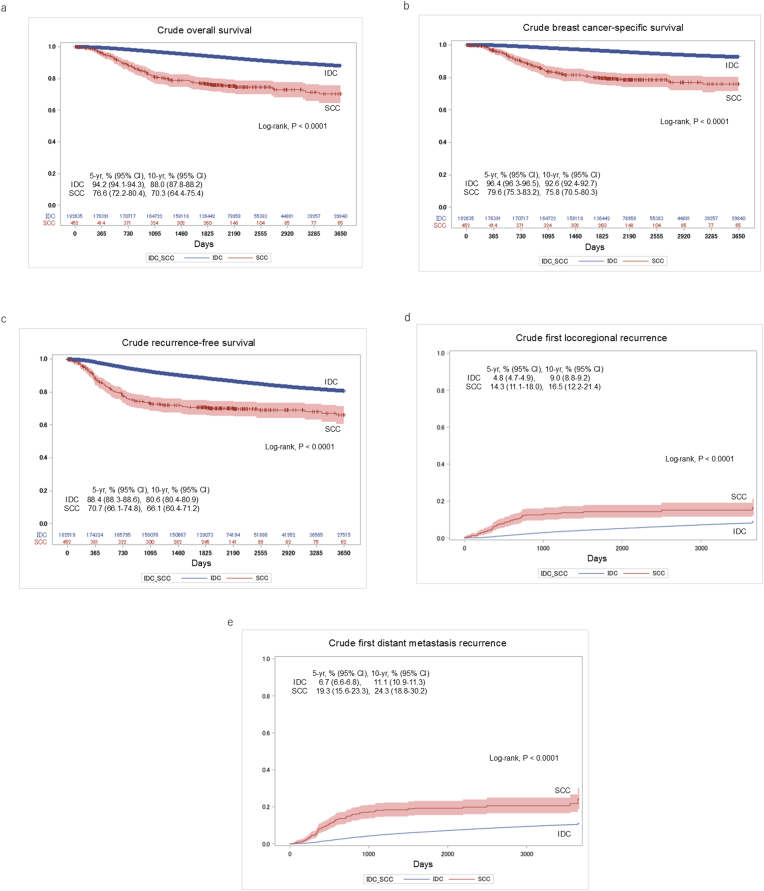
Crude comparisons of (a) overall survival, (b) breast cancer-specific survival, (c) recurrence-free survival, (d) cumulative incidence of first locoregional recurrence, and (e) cumulative incidence of first distant metastasis between SCC and IDC patients in the entire cohort. SCC: squamous cell carcinoma; IDC: invasive ductal carcinoma.

Fig. 2 shows the crude clinical outcomes. The 5- and 10-year OS rates were 76.6 % (95 % CI, 72.2–80.4) and 70.3 % (64.4–75.4) for SCC, and 94.2 % (94.1–94.3) and 88.0 % (87.8–88.2) for IDC, respectively (Fig. 2a). The 5- and 10-year BCSS rates were 79.6 % (95 % CI, 75.3–83.2) and 75.8 % (70.5–80.3) for SCC, and 96.4 % (96.3–96.5) and 92.6 % (92.4–92.7) for IDC, respectively (Fig. 2b). The 5- and 10-year RFS rates were 70.7 % (95 % CI, 66.1–74.8) and 66.1 % (60.4–71.2) for SCC, and 88.4 % (88.3–88.6) and 80.6 % (80.4–80.9) for IDC, respectively (Fig. 2c). The cumulative incidences of first locoregional recurrence at 5 and 10 years were 14.3 % (95 % CI, 11.1–18.0) and 16.5 % (12.2–21.4) for SCC, and 4.8 % (4.7–4.9) and 9.0 % (8.8–9.2) for IDC, respectively (Fig. 2d). The cumulative incidences of first distant metastasis recurrence at 5 and 10 years were 19.3 % (95 % CI, 15.6–23.3) and 24.3 % (18.8–30.2) for SCC, and 6.7 % (6.6–6.8) and 11.1 % (10.9–11.3) for IDC, respectively (Fig. 2e).

### 3.3 Clinical outcomes of patients with the triple-negative subtype after exact matching

**Fig. 3:**
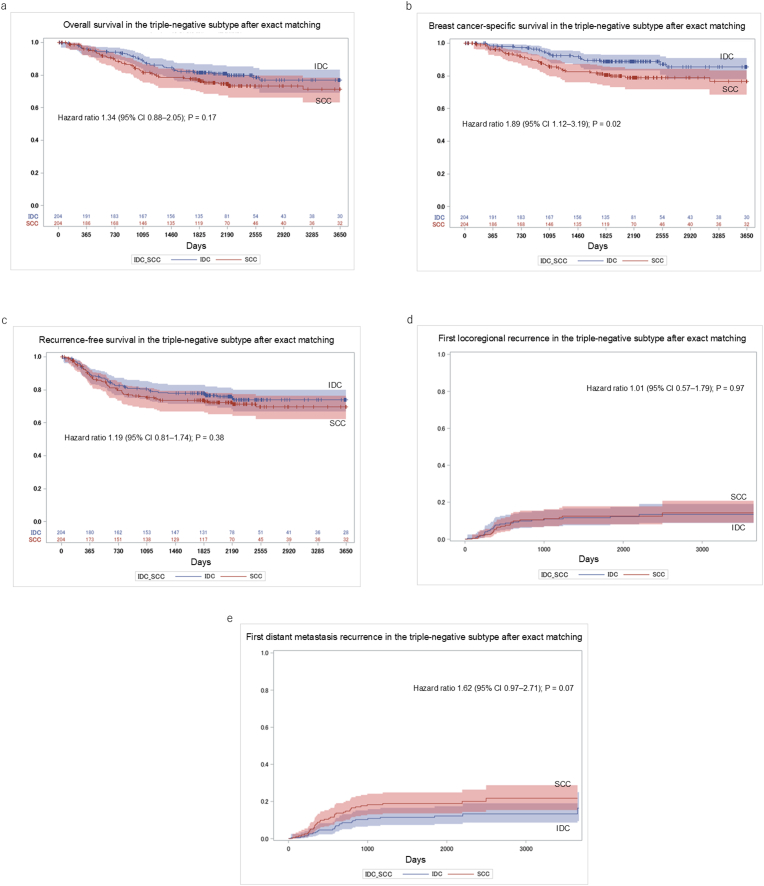
Survival and recurrence outcomes in the triple-negative subtype after exact matching between SCC and IDC patients: (a) overall survival, (b) breast cancer-specific survival, (c) recurrence-free survival, (d) cumulative incidence of first locoregional recurrence, and (e) cumulative incidence of first distant metastasis. SCC: squamous cell carcinoma; IDC: invasive ductal carcinoma.

Among patients with the triple-negative subtype after exact matching (204 in each group), the 5- and 10-year OS rates were 76.8 % and 71.4 % for SCC versus 81.6 % and 76.9 % for IDC; this difference was not statistically significant (HR 1.34, 95 % CI 0.88–2.05; P = 0.17; Fig. 3a). The 5- and 10-year BCSS rates were 80.5 % and 76.7 % for SCC compared to 88.8 % and 85.5 % for IDC, indicating a significantly worse BCSS for SCC (HR 1.89, 95 % CI 1.12–3.19; P = 0.02, Fig. 3b). RFS rates at 5 and 10 years were 73.6 % vs 77.8 % and 69.7 % vs 73.9 %, respectively (HR 1.19, 95 % CI 0.81–1.74; P = 0.38; Fig. 3c), showing no significant difference. The cumulative incidence of first locoregional recurrence at 5 and 10 years was 12.4 % and 14.2 % for SCC versus 11.6 % and 13.4 % for IDC (HR 1.01, 95 % CI 0.57–1.79; P = 0.97, Fig. 3d), while first distant metastasis occurred in 18.9 % and 21.7 % of SCC versus 11.5 % and 16.4 % for IDC (HR 1.62, 95 % CI 0.97–2.71; P = 0.07; Fig. 3e), neither reaching statistical significance.

### 3.4 Clinical outcomes of patients with the luminal subtype after exact matching

**Fig. 4:**
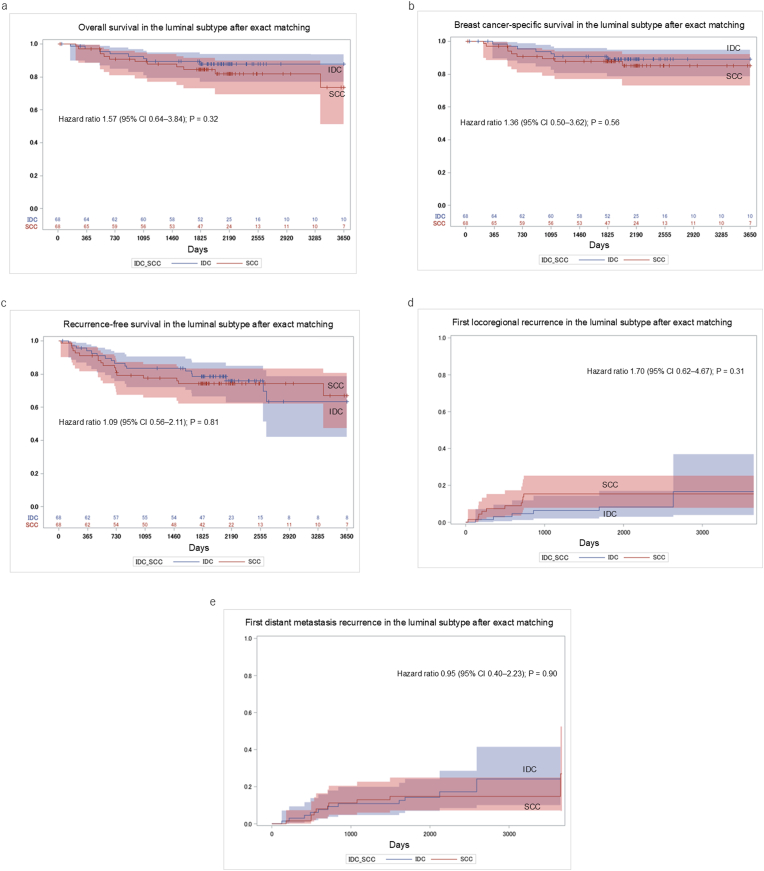
Survival and recurrence outcomes in the luminal subtype after exact 1:1 matching between SCC and IDC patients: (a) overall survival, (b) breast cancer-specific survival, (c) recurrence-free survival, (d) cumulative incidence of first locoregional recurrence, and (e) cumulative incidence of first distant metastasis. SCC: squamous cell carcinoma; IDC: invasive ductal carcinoma.

Among the luminal subtype cohort (68 patients per group), no significant differences between SCC and IDC were observed after exact matching across all survival and recurrence endpoints. At 5 and 10 years, OS rates were 84.3 % and 73.7 % for SCC, compared to 87.8 % for IDC at both intervals (HR 1.57; 95 % CI 0.64–3.84; P = 0.32; Fig. 4a). BCSS rates were 87.6 % and 85.1 % for SCC, versus 89.1 % for IDC at both time points (HR 1.36; 95 % CI 0.50–3.62; P = 0.56; Fig. 4b). RFS rates at 5 and 10 years were 74.4 % and 67.0 % for SCC and 78.6 % and 63.2 % for IDC (HR 1.09; 95 % CI 0.56–2.11; P = 0.81; Fig. 4c).The cumulative incidence of first locoregional recurrence at 5 and 10 years was 15.4 % for SCC, compared to 8.3 % and 16.6 % for IDC (HR 1.70; 95 % CI 0.62–4.67; P = 0.31; Fig. 4d). The incidence of first distant metastasis was 14.8 % and 27.0 % for SCC, compared to 14.3 % and 24.2 % for IDC, respectively (HR 0.95; 95 % CI 0.40–2.23; P = 0.90; Fig. 4e).

### 3.5 In-field area recurrence after adjuvant radiotherapy: crude and exact-matched analyses

**Fig. 5:**
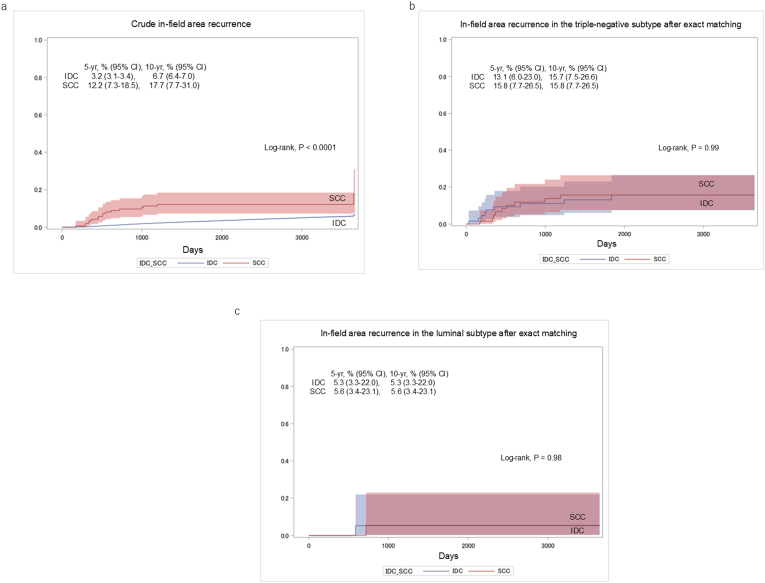
Cumulative incidence of in-field area recurrence following adjuvant radiotherapy, comparing SCC and IDC: (a) crude analysis of the full cohort; (b) triple-negative subtype after exact matching; (c) luminal subtype after exact matching. SCC: squamous cell carcinoma; IDC: invasive ductal carcinoma.

Crude in-field area recurrence was greater in SCC than in IDC (P < 0.001; Fig. 5a). After exact matching (65 pairs for triple-negative and 19 for luminal subtypes), no significant differences remained. In the triple-negative subtype, the cumulative incidences of in-field area recurrence at 5 and 10 years were both 15.8 % for SCC and 13.1 % and 15.7 % for IDC (P = 0.99; Fig. 5b). In the luminal subtype, 5- and 10- years cumulative in-field area recurrence rates were both 5.6 % for SCC and both 5.3 % for IDC (P = 0.98; Fig. 5c).

### 3.6 Sensitivity analysis via PSM

To assess the robustness of our findings, we conducted a sensitivity analysis via PSM with the same covariates as used in exact matching. In the triple-negative subtype, PSM confirmed the results obtained from exact matching—BCSS remained significantly worse in SCC, while OS, RFS, and recurrence outcomes showed no significant differences. However, in the luminal subtype, outcomes diverged: exact matching showed no significant differences between SCC and IDC across all endpoints, but the PSM sensitivity analysis revealed statistically significant differences in BCSS, RFS, first locoregional recurrence, and first distant metastasis. PSM results are presented in Supplementary Table 4 and Supplementary Figs. 1 and 2.

## 4 Discussion

In this study, we compared the clinical characteristics and prognosis between SCC and IDC using data from the NCD-BCR, the largest breast cancer registry in Japan. We observed that SCC was more frequently diagnosed at an advanced stage and was associated with worse outcomes. SCC histology was an independent predictor of poor prognosis in the triple-negative subtype. Among patients who received adjuvant radiotherapy, no significant difference was found in the in-field area recurrence rate between SCC and IDC after exact matching. To our knowledge, this represents the most extensive investigation of breast SCC in an Asian population to date.

In support of our findings, previous large-scale registry studies have showed the characteristics of SCC. Zhu et al. analyzed 686 SCC cases from the U.S. National Cancer Database and reported high rates of ER-negative (74.9 %), PgR-negative (87.8 %), and HER2-negative (87.7 %), with nodal involvement in 17.9 % [16]. The 5- and 10-year OS rates were 62.1 % and 50.6 %, respectively. Similarly, several studies using the National Cancer Institute Surveillance, Epidemiology, and End Results Registry have reported ER/PgR negativity in 57.5–63.5 % and nodal involvement in 20.0–30.8 % [12,17,20]. The 5- and 10-year OS rates were 63.5–77.0 % and 77 %, respectively. These results are consistent with our Japanese database results and suggest that the clinical and pathological characteristics of SCC are similar across populations.

In our study, 58.8 % of patients with SCC had the triple-negative subtype, whereas only 10.0 % of patients with IDC did. Since TNBC is clinically aggressive and has a poor prognosis [18,19], we conducted subtype-specific comparisons, adjusting for clinical factors, to clarify whether the poorer prognosis of SCC was attributable to the high incidence of TNBC or to the SCC histology itself. We found that patients with SCC had a significantly worse BCSS than those with IDC did. Our primary analyses rely on exact 1:1 matching, which ensures perfect balance across all matched covariates and limits confounding. In the triple-negative subtype, PSM confirmed these results, reinforcing their robustness. In the luminal subtype, however, PSM revealed significant differences that exact matching did not detect. Smaller sample size of exact matching may have reduced statistical power, and PSM may allow slight imbalance in covariates while retaining more cases. Consequently, although the PSM findings raise important hypotheses regarding SCC in the luminal subtype, they should be interpreted with caution.

To our knowledge, no previous studies have evaluated the prognosis of breast SCC by subtype. However, similar results have been reported for metaplastic breast carcinoma. Retrospective studies have shown that patients with metaplastic breast carcinoma have worse survival and a higher risk of local recurrence when matched to patients with TNBC [21,22]. Another study comparing 47 metaplastic sarcomatoid carcinoma patients with TNBC patients matched by age, clinical stage, and therapeutic modality found worse DFS [23].

In addition to its typical classification as triple-negative, breast SCC presents distinct biological characteristics. One notable feature is the overexpression of epidermal growth factor receptor (EGFR). A previous study reported that 87 % of breast SCC cases were EGFR-positive [24]. In addition, breast SCC has been reported to have a high frequency of TP53 mutations (approximately 85 % of cases) [25]. Mutant p53 not only loses its tumor-suppressing function but also undergoes a gain-of function that promotes tumor progression and metastasis [26]. Both EGFR overexpression and TP53 mutation are strongly associated with advanced malignancies and poor prognosis [[26], [27], [28]], and may help explain the poorer outcomes in SCC.

Moreover, a lower complete pathological response rate to anthracycline- and taxane-based chemotherapy has been reported in metaplastic breast carcinoma, which includes SCC as a histological subtype, than in other TNBC [25]. In our cohort, the most frequently used chemotherapy regimen was a combination of anthracyclines and taxanes. This standard chemotherapy may be insufficiently effective in the treatment of for SCC because of chemoresistance, resulting in a poor prognosis. To overcome chemoresistance in SCC, targeted therapies, such as EGFR inhibitors or TP53-directed agents, may offer clinical benefit.

The efficacy of adjuvant radiotherapy for SCC is unknown. A previous study demonstrated that adjuvant radiotherapy after BCS significantly improved survival compared with BCS alone in SCC patients [12]. Because adjuvant radiotherapy reduces breast cancer mortality and is a widely used standard treatment [29], the therapeutic effect of this approach is expected to be similar in SCC. However, a multicenter retrospective cohort study indicated that SCC had a greater incidence of locoregional recurrence after adjuvant radiotherapy, particularly in-field recurrence [13]. Our study revealed that in-field area recurrence was greater in SCC patients before matching, but there were no significant differences in in-field area recurrence after matching. These finidings suggest that the local control achieved by radiotherapy in SCC patients may be comparable to that in IDC patients. Adjuvant radiotherapy could be considered a reasonable treatment option for the management of SCC.

Our study has several limitations. As no central pathology review was conducted, heterogeneity in SCC diagnosis cannot be completely eliminated. Although our sample size was relatively large, the sample size for each subtype was small, limiting statistical power. Given the rarity of HER2-positive SCC, the number of patients in our cohort was inadequate to perform meaningful statistical comparisons. Future investigations with larger sample sizes are needed to clarify the clinical characteristics and outcomes of HER2-positive SCC. The lack of detailed information on chemotherapy schedules was another limitation. Although the types of chemotherapy agents were availabe, the intensity of chemotherapy also influences cancer prognosis. Moreover, the lack of information on the actual irradiated fields was an important limitation. As a substitute endpoint, we used in-field area recurrence. However, this measure does not perfectly reflect true in-field recurrence, as we could not confirm whether the recurrence occurred within the precise irradiated field. Additionally, although we used exact matching, residual confounding remains possible owing to the limitations of registry-based data. The imbalance in TNBC prevalence between SCC and IDC and the molecular heterogeneity within TNBC may have influenced the outcomes. To address this possibility, we conducted a sensitivity analysis via PSM, yet these limitations remain important considerations when interpreting our findings.

Despite these limitations, our study is the first to examine the prognosis of SCC and the effects of radiotherapy by subtype using a large series of real-world data with matching background factors. Further study is needed to clarify the optimal treatment for SCC.

## 5 Conclusion

Breast SCC was often diagnosed at an advanced stage and had a worse prognosis than IDC. SCC histology was found to be an independent factor for a worse prognosis in the triple-negative subtype. In-filed area recurrence appeared to be comparable between SCC and IDC, although this finding should be interpreted with caution owing to limitations in the definition of the irradiated field.

## CRediT authorship contribution statement

**Mami Ogita:** Writing – original draft, Visualization, Project administration, Methodology, Investigation, Formal analysis, Conceptualization. **Hiraku Kumamaru:** Writing – review & editing, Resources, Methodology, Investigation, Formal analysis, Data curation, Conceptualization. **Makoto Kubo:** Writing – review & editing, Project administration, Methodology, Investigation, Formal analysis, Conceptualization. **Naoko Kinukawa:** Writing – review & editing, Visualization, Formal analysis, Data curation, Conceptualization. **Naoki Niikura:** Writing – review & editing, Supervision, Conceptualization. **Shigehira Saji:** Writing – review & editing, Supervision, Conceptualization. **Masakazu Toi:** Writing – review & editing, Supervision, Conceptualization.

## Ethics approval and consent to participate

The study was conducted in accordance with the relevant guidelines and local regulations and was approved by the Institutional Review Board of the University of Tokyo Hospital (2020080NI). Because this study was a retrospective analysis using deidentified data from a national registry, the requirement for informed consent was waived by the ethics committee.

## Data availability

The analytic data are not publicly available owing to the data use policy of the NCD.

## Funding

This research was supported by the Japanese Breast Cancer Society. The funding body had no role in the study design, collection, analysis, or interpretation of the data or the writing of the manuscript.

## Declaration of comepting interst

HK reports receiving consultation fees from EPS Corporation, speaker fees from Chugai Pharmaceutical Co., Ltd, and a research grant from Amgen K.K. for an unrelated study topic. HK is affiliated with the Department of Healthcare Quality Assessment at the University of Tokyo, a social collaboration department supported by the National Clinical Database, Johnson & Johnson K.K., Nipro Corporation, and Intuitive Surgical Sàrl.

NK is affiliated with the Department of Healthcare Quality Assessment at the University of Tokyo, a social collaboration department supported by the National Clinical Database, Johnson & Johnson K.K., Nipro Corporation, and Intuitive Surgical Sàrl.

SS reports lecture fees from 10.13039/100010795Chugai, 10.13039/501100004095Kyowa Kirin, 10.13039/100030732MSD, Novartis, Eisai, Takeda, Daiichi Sankyo, Eli Lilly, Astra Zeneca, Pfizer, Taiho, Ono, Nipponkayaku, Gilead, and Exact Sciences, advisory role for Chugai/Roche, Astra Zeneca, Eli Lilly, Pfizer, Kyowa Kirin, Daiichi Sankyo, and MSD, research grants from Taiho, Eisai, Chugai, Takeda, MSD, Astra Zeneca, Daiichi Sankyo, Gilead, Eli Lilly, Sanofi and Executive board member of JBCRG, JBCS, JSMO and BIG.

MT reports research grants from Chugai, Takeda, Pfizer, Taiho, JBCRG assoc., KBCRN assoc., Eisai, Eli-Lilly and companies, 10.13039/501100002973Daiichi-Sankyo, 10.13039/100004325AstraZeneca, 10.13039/501100004948Astellas, 10.13039/100016846Shimadzu, 10.13039/501100012030Yakult, 10.13039/100018046Nippon Kayaku, AFI technology, Luxonus, Shionogi, GL Science, and Sanwa Shurui, lecture honoraria or lecture chair from Chugai, Takeda, Pfizer, Kyowa-Kirin, Taiho, Eisai, Daiichi-Sankyo, AstraZeneca, Eli Lilly and companies, MSD, Exact Science, Novartis, 10.13039/100016846Shimadzu, 10.13039/501100012030Yakult, 10.13039/100018046Nippon Kayaku, Devicore Medical Japan, and Sysmex, participation on an advisory board of Daiichi-Sankyo, Eli Lilly and companies, BMS, Bertis, Terumo, Kansai Medical Net, member of the board of directors (no salary) of Assoc. JBCRG, Assoc. KBCRN, NPO org. OOTR, Chairman of the board of directors (no salary) of Assoc. JBCS, and Associate editor of British Journal of Cancer, Scientific Reports, Breast Cancer Research and Treatment, Cancer Science, Asian Journal of Surgery, Asian Journal of Breast Surgery.

The other authors declare that they have no conflicts of interest associated with this manuscript.
