# Supplementary material for: Survival of patients with squamous cell carcinoma of the breast compared with invasive ductal carcinoma by biological subtype: A matched analysis of the Japanese national clinical database-breast cancer registry
Source: Breast. 2025 Aug 27;83:104567. doi: 10.1016/j.breast.2025.104567 (PMC12433503; doi:10.1016/j.breast.2025.104567)
Supplement: Multimedia component 1 [file mmc1.docx]

Supplementary

Supplementary Table 1

Type of neoadjuvant and adjuvant chemotherapy and information of adjuvant radiotherapy for stage I-III SCC and IDC patients starting treatment in 2004-2014 in NCD-BC

|  |  | SCC |  | IDC |  |
| --- | --- | --- | --- | --- | --- |
| Type of neoadjuvant chemotherapy |  | n=109 | % | n=25,053 | % |
|  | CMF | 0 | 0.0 | 16 | 0.1 |
|  | Anthracyclines | 19 | 17.4 | 3,388 | 13.5 |
|  | Taxane | 17 | 15.6 | 2,837 | 11.3 |
|  | Anthracyclines + taxane | 71 | 65.1 | 18,093 | 72.2 |
|  | Others | 2 | 1.8 | 712 | 2.8 |
|  | Unknown | 0 | 0.0 | 7 | 0.0 |
| Type of adjuvant chemotherapy |  | n=266 | % | n=58,328 | % |
|  | CMF | 4 | 1.5 | 2,078 | 3.6 |
|  | Anthracyclines | 74 | 27.8 | 17,501 | 30.0 |
|  | Taxane | 61 | 22.9 | 12,860 | 22.0 |
|  | Anthracyclines + taxane | 82 | 30.8 | 19,888 | 34.1 |
|  | Others | 45 | 16.9 | 5,994 | 10.3 |
|  | Unknown | 0 | 0.0 | 7 | 0.0 |
| Irradiated area of adjuvant radiotherapy |  | n=148 | % | n=88,840 | % |
|  | Breast | 95 | 64.2 | 75,236 | 84.7 |
|  | Chest wall | 16 | 10.8 | 2,949 | 3.3 |
|  | Breast + RNI | 5 | 3.4 | 3,917 | 4.4 |
|  | Chest wall + RNI | 31 | 20.9 | 5,769 | 6.5 |
|  | Others | 1 | 0.7 | 964 | 1.1 |
|  | Unknown | 0 | 0.0 | 5 | 0.0 |

SCC: squamous cell carcinoma; IDC: invasive ductal carcinoma; CMF: cyclophosphamide endoxan fluorouracil; RNI: regional nodal irradiation

Supplementary Table 2

The SCC and IDC patient characteristics and treatment pattern of triple-negative and luminal subtypes before matching

|  |  | Triple-negative | | | |  | Luminal | | | |  |
| --- | --- | --- | --- | --- | --- | --- | --- | --- | --- | --- | --- |
|  |  | SCC |  | IDC |  |  | SCC |  | IDC |  |  |
|  |  | n=253 | % | n= 17,416 | % | SMD | n=83 | % | n= 10,8364 | % | SMD |
| Year of surgery | 2004-06 | 41 | 16.2 | 2,873 | 16.5 | 0.05 | 12 | 14.5 | 14,489 | 13.4 | 0.27 |
|  | 2007-09 | 58 | 22.9 | 4,363 | 25.1 |  | 11 | 13.3 | 25,498 | 23.5 |  |
|  | 2010-12 | 103 | 40.7 | 6,751 | 38.8 |  | 39 | 47.0 | 44,161 | 40.8 |  |
|  | 2013-14 | 51 | 20.2 | 3,429 | 19.7 |  | 21 | 25.3 | 24,216 | 22.3 |  |
| Age, year | -29 | 1 | 0.4 | 153 | 0.9 | 0.16 | 2 | 2.4 | 385 | 0.4 | 0.19 |
|  | 30-39 | 15 | 5.9 | 1,380 | 7.9 |  | 4 | 4.8 | 6,288 | 5.8 |  |
|  | 40-49 | 34 | 13.4 | 2,849 | 16.4 |  | 20 | 24.1 | 25,656 | 23.7 |  |
|  | 50-59 | 71 | 28.1 | 4,253 | 24.4 |  | 18 | 21.7 | 23,800 | 22.0 |  |
|  | 60-69 | 64 | 25.3 | 4,631 | 26.6 |  | 22 | 26.5 | 27,602 | 25.5 |  |
|  | 70- | 68 | 26.9 | 4,150 | 23.8 |  | 17 | 20.5 | 24,633 | 22.7 |  |
| cT | T1 | 40 | 15.8 | 6,886 | 39.5 | 0.64 | 16 | 19.3 | 64,752 | 59.8 | 0.96 |
|  | T2 | 138 | 54.5 | 8,468 | 48.6 |  | 47 | 56.6 | 36,560 | 33.7 |  |
|  | T3 | 26 | 10.3 | 1,003 | 5.8 |  | 12 | 14.5 | 2,778 | 2.6 |  |
|  | T4 | 49 | 19.4 | 1,059 | 6.1 |  | 8 | 9.6 | 4,274 | 3.9 |  |
| cN | N0 | 165 | 65.2 | 11,978 | 68.8 | 0.10 | 50 | 60.2 | 89,275 | 82.4 | 0.51 |
|  | N1 | 70 | 27.7 | 4,297 | 24.7 |  | 28 | 33.7 | 16,475 | 15.2 |  |
|  | N2 | 13 | 5.1 | 700 | 4.0 |  | 4 | 4.8 | 1,730 | 1.6 |  |
|  | N3 | 5 | 2.0 | 441 | 2.5 |  | 1 | 1.2 | 884 | 0.8 |  |
| cStage | I | 34 | 13.4 | 5,958 | 34.2 |  | 14 | 16.9 | 60,009 | 55.4 |  |
|  | II | 183 | 72.3 | 10,498 | 60.3 |  | 60 | 72.3 | 45,061 | 41.6 |  |
|  | III | 36 | 14.2 | 960 | 5.5 |  | 9 | 10.8 | 3,294 | 3.0 |  |
| Surgery | BCS | 83 | 32.8 | 9,493 | 54.5 | -0.45 | 24 | 28.9 | 66,864 | 61.7 | -0.70 |
|  | Mastectomy | 170 | 67.2 | 7,923 | 45.5 |  | 59 | 71.1 | 41,500 | 38.3 |  |
| Axillary surgery | SLNB | 99 | 39.1 | 7,500 | 43.1 | 0.12 | 24 | 28.9 | 58,082 | 53.6 | 0.52 |
|  | ALND | 137 | 54.2 | 9,172 | 52.7 |  | 55 | 66.3 | 45,410 | 41.9 |  |
|  | No | 17 | 6.7 | 744 | 4.3 |  | 4 | 4.8 | 4,872 | 4.5 |  |
| ER | Positive | 0 | 0.0 | 0 | 0.0 |  | 78 | 94.0 | 107,149 | 98.9 |  |
|  | Negative | 253 | 100.0 | 17,416 | 100.0 |  | 5 | 6.0 | 1,201 | 1.1 |  |
|  | Unknown | 0 | 0.0 | 0 | 0.0 |  | 0 | 0.0 | 13 | 0.0 |  |
| PgR | Positive | 0 | 0.0 | 0 | 0.0 |  | 31 | 37.3 | 92,604 | 85.5 |  |
|  | Negative | 253 | 100.0 | 17,416 | 100.0 |  | 51 | 61.4 | 15,547 | 14.3 |  |
|  | Unknown | 0 | 0.0 | 0 | 0.0 |  | 1 | 1.2 | 213 | 0.2 |  |
| HER2 | Positive | 0 | 0.0 | 0 | 0.0 |  | 0 | 0.0 | 0 | 0.0 |  |
|  | Negative | 253 | 100.0 | 17,416 | 100.0 |  | 83 | 100.0 | 108,364 | 100.0 |  |
| Neoadjuvant chemotherapy | Yes | 58 | 22.9 | 4,454 | 25.6 | -0.06 | 22 | 26.5 | 9,987 | 9.2 | 0.46 |
|  | No | 195 | 77.1 | 12,962 | 74.4 |  | 61 | 73.5 | 98,377 | 90.8 |  |
| Type | CMF | 0 | 0.0 | 3 | 0.0 |  | 0 | 0.0 | 4 | 0.0 |  |
|  | Anthracyclines | 11 | 19.0 | 622 | 14.0 |  | 3 | 13.6 | 1,322 | 13.2 |  |
|  | Taxane | 7 | 12.0 | 317 | 7.1 |  | 3 | 13.6 | 973 | 9.7 |  |
|  | Anthracyclines + taxane | 38 | 65.5 | 3,430 | 77.0 |  | 16 | 72.7 | 7,376 | 73.9 |  |
|  | Others | 2 | 3.4 | 82 | 1.8 |  | 0 | 0.0 | 312 | 3.1 |  |
| Neoadjuvant molecular targeted therapy | Yes | 3 | 1.2 | 95 | 0.5 | 0.07 | 0 | 0.0 | 253 | 0.2 | -0.07 |
|  | No | 250 | 98.8 | 17,321 | 99.5 |  | 83 | 100.0 | 108,111 | 99.8 |  |
| Neoadjuvant endocrine therapy | Yes | 3 | 1.2 | 117 | 0.7 | 0.05 | 1 | 1.2 | 4,670 | 4.3 | -0.19 |
|  | No | 250 | 98.8 | 17,299 | 99.3 |  | 82 | 98.8 | 103,694 | 95.7 |  |
| Adjuvant chemotherapy | Yes | 156 | 61.7 | 9,966 | 57.2 | 0.09 | 44 | 53.0 | 26,053 | 24.0 | 0.62 |
|  | No | 97 | 38.3 | 7,450 | 42.8 |  | 39 | 47.0 | 82,311 | 76.0 |  |
| Type | CMF | 4 | 2.6 | 407 | 4.1 |  | 0 | 0.0 | 974 | 3.7 |  |
|  | Anthracyclines | 42 | 26.9 | 2,966 | 29.8 |  | 12 | 27.3 | 6,933 | 26.6 |  |
|  | Taxane | 37 | 23.7 | 1,786 | 17.9 |  | 9 | 20.5 | 6,654 | 25.5 |  |
|  | Anthracyclines + taxane | 44 | 28.2 | 3,326 | 33.4 |  | 18 | 40.9 | 9,083 | 34.9 |  |
|  | Others | 29 | 18.6 | 1,479 | 14.8 |  | 5 | 11.4 | 2,409 | 9.2 |  |
|  | Unkown | 0 | 0.0 | 2 | 0.0 |  | 0 | 0.0 | 0 | 0.0 |  |
| Adjuvant molecular targeted therapy | Yes | 2 | 0.8 | 235 | 1.3 | -0.05 | 2 | 2.4 | 630 | 0.6 | 0.15 |
|  | No | 251 | 99.2 | 17,181 | 98.7 |  | 81 | 97.6 | 107,734 | 99.4 |  |
| Adjuvant endocrine therapy | Yes | 5 | 2.0 | 908 | 5.2 | -0.17 | 48 | 57.8 | 96,064 | 88.6 | -0.74 |
|  | No | 248 | 98.0 | 16,508 | 94.8 |  | 35 | 42.2 | 12,300 | 11.4 |  |
| Adjuvant radiotherapy | Yes | 86 | 34.0 | 6,977 | 40.1 | -0.13 | 27 | 32.5 | 57,981 | 53.5 | -0.43 |
|  | No | 167 | 66.0 | 10,439 | 59.9 |  | 56 | 67.5 | 50,383 | 46.5 |  |
| Irradiated site | Breast | 56 | 65.1 | 5,418 | 77.7 |  | 16 | 59.3 | 49,472 | 85.3 |  |
|  | Chest wall | 9 | 10.5 | 236 | 3.4 |  | 2 | 7.4 | 1,100 | 1.9 |  |
|  | Breast + RNI | 2 | 2.3 | 372 | 5.3 |  | 2 | 7.4 | 2,436 | 4.2 |  |
|  | Chest wall + RNI | 16 | 18.6 | 623 | 8.9 |  | 6 | 22.2 | 3,215 | 5.5 |  |
|  | Others | 3 | 3.5 | 327 | 4.7 |  | 1 | 3.7 | 1,754 | 3.0 |  |
|  | Unknown | 0 | 0.0 | 1 | 0.0 |  | 0 | 0.0 | 4 | 0.0 |  |

SCC: squamous cell carcinoma; IDC: invasive ductal carcinoma; SMD: standardized mean difference; BCS: breast-conserving surgery; SLNB: sentinel lymph node biopsy; ALND: axillary lymph node dissection; ER: estrogen receptor; PgR: progesterone receptor; HER2: human epidermal growth factor receptor 2; CMF: cyclophosphamide endoxan fluorouracil; RNI: regional nodal irradiation

Supplementary Table 3

The SCC and IDC patient characteristics and treatment pattern of triple-negative and luminal subtypes after exact matching

|  |  | Triple-negative | | | | Luminal | | | |
| --- | --- | --- | --- | --- | --- | --- | --- | --- | --- |
|  |  | SCC |  | IDC |  | SCC |  | IDC |  |
|  |  | n=204 | % | n=204 | % | n=68 | % | n=68 | % |
| Year of surgery | 2004-06 | 30 | 14.7 | 30 | 14.7 | 11 | 16.2 | 11 | 16.2 |
|  | 2007-09 | 53 | 26.0 | 53 | 26.0 | 8 | 11.8 | 8 | 11.8 |
|  | 2010-12 | 83 | 40.7 | 83 | 40.7 | 33 | 48.5 | 33 | 48.5 |
|  | 2013-14 | 38 | 18.6 | 38 | 18.6 | 16 | 23.5 | 16 | 23.5 |
| Age, year | -29 | 0 | 0.0 | 0 | 0.0 | 1 | 1.5 | 0 | 0.0 |
|  | 30-39 | 11 | 5.4 | 12 | 5.9 | 3 | 4.4 | 4 | 5.9 |
|  | 40-49 | 24 | 11.8 | 21 | 10.3 | 18 | 26.5 | 18 | 26.5 |
|  | 50-59 | 56 | 27.5 | 58 | 28.4 | 14 | 20.6 | 13 | 19.1 |
|  | 60-69 | 54 | 26.5 | 53 | 26.0 | 19 | 27.9 | 19 | 27.9 |
|  | 70- | 59 | 28.9 | 60 | 29.4 | 13 | 19.1 | 14 | 20.6 |
| cT | T1 | 34 | 16.7 | 34 | 16.7 | 15 | 22.1 | 15 | 22.1 |
|  | T2 | 126 | 61.8 | 126 | 61.8 | 41 | 60.3 | 41 | 60.3 |
|  | T3 | 21 | 10.3 | 21 | 10.3 | 9 | 13.2 | 9 | 13.2 |
|  | T4 | 23 | 11.3 | 23 | 11.3 | 3 | 4.4 | 3 | 4.4 |
| cN | N0 | 141 | 69.1 | 141 | 69.1 | 43 | 63.2 | 43 | 63.2 |
|  | N1 | 54 | 26.5 | 54 | 26.5 | 24 | 35.3 | 24 | 35.3 |
|  | N2 | 8 | 3.9 | 8 | 3.9 | 1 | 1.5 | 1 | 1.5 |
|  | N3 | 1 | 0.5 | 1 | 0.5 | 0 | 0.0 | 0 | 0.0 |
| cStage | I | 31 | 15.2 | 31 | 15.2 | 14 | 20.6 | 14 | 20.6 |
|  | II | 156 | 76.5 | 155 | 76.0 | 49 | 72.1 | 49 | 72.1 |
|  | III | 17 | 8.3 | 18 | 8.8 | 5 | 7.4 | 5 | 7.4 |
| Surgery | BCS | 69 | 33.8 | 69 | 33.8 | 23 | 33.8 | 23 | 33.8 |
|  | Mastectomy | 135 | 66.2 | 135 | 66.2 | 45 | 66.2 | 45 | 66.2 |
| Axillary surgery | SLNB | 89 | 43.6 | 89 | 43.6 | 21 | 30.9 | 21 | 30.9 |
|  | ALND | 108 | 52.9 | 108 | 52.9 | 44 | 64.7 | 44 | 64.7 |
|  | No | 7 | 3.4 | 7 | 3.4 | 3 | 4.4 | 3 | 4.4 |
| ER | Positive | 0 | 0.0 | 0 | 0.0 | 64 | 94.1 | 65 | 95.6 |
|  | Negative | 204 | 100.0 | 204 | 100.0 | 4 | 5.9 | 3 | 4.4 |
| PgR | Positive | 0 | 0.0 | 0 | 0.0 | 25 | 36.8 | 59 | 86.8 |
|  | Negative | 204 | 100.0 | 204 | 100.0 | 42 | 61.8 | 9 | 13.2 |
|  | Unknown | 0 | 0.0 | 0 | 0.0 | 1 | 1.5 | 0 | 0.0 |
| HER2 | Positive | 0 | 0.0 | 0 | 0.0 | 0 | 0.0 | 0 | 0.0 |
|  | Negative | 204 | 100.0 | 204 | 100.0 | 68 | 100.0 | 68 | 100.0 |
| Neoadjuvant chemotherapy | Yes | 37 | 18.1 | 37 | 18.1 | 12 | 17.6 | 12 | 17.6 |
|  | No | 167 | 81.9 | 167 | 81.9 | 56 | 82.4 | 56 | 82.4 |
| Type | CMF | 0 | 0.0 | 0 | 0.0 | 0 | 0.0 | 0 | 0.0 |
|  | Anthracyclines | 5 | 13.5 | 10 | 27.0 | 3 | 25.0 | 0 | 0.0 |
|  | Taxane | 2 | 5.4 | 3 | 8.1 | 1 | 8.3 | 1 | 8.3 |
|  | Anthracyclines + taxane | 30 | 81.1 | 24 | 64.9 | 8 | 66.7 | 11 | 91.7 |
| Neoadjuvant molecular targeted therapy | Yes | 0 | 0.0 | 0 | 0.0 | 0 | 0.0 | 0 | 0.0 |
|  | No | 204 | 100.0 | 204 | 100.0 | 68 | 100.0 | 68 | 100.0 |
| Neoadjuvant endocrine therapy | Yes | 0 | 0.0 | 0 | 0.0 | 0 | 0.0 | 0 | 0.0 |
|  | No | 204 | 100.0 | 204 | 100.0 | 68 | 100.0 | 68 | 100.0 |
| Adjuvant chemotherapy | Yes | 128 | 62.7 | 128 | 62.7 | 36 | 52.9 | 36 | 52.9 |
|  | No | 76 | 37.3 | 76 | 37.3 | 32 | 47.1 | 32 | 47.1 |
| Type | CMF | 4 | 3.1 | 5 | 3.9 | 0 | 0.0 | 1 | 2.8 |
|  | Anthracyclines | 35 | 27.3 | 38 | 29.7 | 10 | 27.8 | 13 | 36.1 |
|  | Taxane | 27 | 21.1 | 24 | 18.8 | 7 | 19.4 | 10 | 27.8 |
|  | Anthracyclines + taxane | 40 | 31.3 | 44 | 34.4 | 16 | 44.4 | 9 | 25.0 |
|  | Others | 22 | 17.2 | 17 | 13.3 | 3 | 8.3 | 3 | 8.3 |
| Adjuvant molecular targeted therapy | Yes | 0 | 0.0 | 0 | 0.0 | 0 | 0.0 | 0 | 0.0 |
|  | No | 204 | 100.0 | 204 | 100.0 | 68 | 100.0 | 68 | 100.0 |
| Adjuvant endocrine therapy | Yes | 1 | 0.5 | 1 | 0.5 | 43 | 63.2 | 43 | 63.2 |
|  | No | 203 | 99.5 | 203 | 99.5 | 25 | 36.8 | 25 | 36.8 |
| Adjuvant radiotherapy | Yes | 65 | 31.9 | 65 | 31.9 | 19 | 27.9 | 19 | 27.9 |
|  | No | 139 | 68.1 | 139 | 68.1 | 49 | 72.1 | 49 | 72.1 |
| Irradiated site | Breast | 51 | 78.5 | 49 | 75.4 | 15 | 78.9 | 13 | 68.4 |
|  | Chest wall | 6 | 9.2 | 6 | 9.2 | 1 | 5.3 | 0 | 0.0 |
|  | Breast + RNI | 1 | 1.5 | 1 | 1.5 | 1 | 5.3 | 3 | 15.8 |
|  | Chest wall + RNI | 7 | 10.8 | 8 | 12.3 | 2 | 10.5 | 3 | 15.8 |
|  | Others | 0 | 0.0 | 1 | 1.5 | 0 | 0.0 | 0 | 0.0 |

SCC: squamous cell carcinoma; IDC: invasive ductal carcinoma; BCS: breast-conserving surgery; SLNB: sentinel lymph　node biopsy; ALND: axillary lymph node dissection; ER: estrogen receptor; PgR: progesterone receptor; HER2: human epidermal growth factor receptor 2; CMF: cyclophosphamide endoxan fluorouracil; RNI: regional nodal irradiation

Supplementary Table 4

The SCC and IDC patient characteristics and treatment pattern of triple-negative and luminal subtypes after propensity score matching

|  |  | Triple-negative | | | |  | Luminal | | | |  |
| --- | --- | --- | --- | --- | --- | --- | --- | --- | --- | --- | --- |
|  |  | SCC |  | IDC |  |  | SCC |  | IDC |  |  |
|  |  | n=253 | % | n= 508 | % | SMD | n=83 | % | n= 164 | % | SMD |
| Year of surgery | 2004-06 | 41 | 16.2 | 71 | 14.0 | 0.09 | 12 | 14.5 | 27 | 16.5 | 0.06 |
|  | 2007-09 | 58 | 22.9 | 132 | 26.0 |  | 11 | 13.3 | 20 | 12.2 |  |
|  | 2010-12 | 103 | 40.7 | 206 | 40.6 |  | 39 | 47.0 | 77 | 47.0 |  |
|  | 2013-14 | 51 | 20.2 | 99 | 19.5 |  | 21 | 25.3 | 40 | 24.4 |  |
| Age, year | -29 | 1 | 0.4 | 4 | 0.8 | 0.05 | 2 | 2.4 | 0 | 0.0 | 0.23 |
|  | 30-39 | 15 | 5.9 | 29 | 5.7 |  | 4 | 4.8 | 9 | 5.5 |  |
|  | 40-49 | 34 | 13.4 | 69 | 13.6 |  | 20 | 24.1 | 41 | 25.0 |  |
|  | 50-59 | 71 | 28.1 | 142 | 28.0 |  | 18 | 21.7 | 38 | 23.2 |  |
|  | 60-69 | 64 | 25.3 | 125 | 24.6 |  | 22 | 26.5 | 46 | 28.0 |  |
|  | 70- | 68 | 26.9 | 139 | 27.4 |  | 17 | 20.5 | 30 | 18.3 |  |
| cT | T1 | 40 | 15.8 | 75 | 14.8 | 0.08 | 16 | 19.3 | 31 | 18.9 | 0.06 |
|  | T2 | 138 | 54.5 | 286 | 56.3 |  | 47 | 56.6 | 90 | 54.9 |  |
|  | T3 | 26 | 10.3 | 60 | 11.8 |  | 12 | 14.5 | 24 | 14.6 |  |
|  | T4 | 49 | 19.4 | 87 | 17.1 |  | 8 | 9.6 | 19 | 11.6 |  |
| cN | N0 | 165 | 65.2 | 326 | 64.2 | 0.09 | 50 | 60.2 | 103 | 62.8 | 0.16 |
|  | N1 | 70 | 27.7 | 152 | 29.9 |  | 28 | 33.7 | 53 | 32.3 |  |
|  | N2 | 13 | 5.1 | 25 | 4.9 |  | 4 | 4.8 | 8 | 4.9 |  |
|  | N3 | 5 | 2.0 | 5 | 1.0 |  | 1 | 1.2 | 0 | 0.0 |  |
| cStage | I | 34 | 13.4 | 65 | 12.8 |  | 14 | 16.9 | 28 | 17.1 |  |
|  | II | 183 | 72.3 | 389 | 76.6 |  | 60 | 72.3 | 118 | 72.0 |  |
|  | III | 36 | 14.2 | 54 | 10.6 |  | 9 | 10.8 | 18 | 11.0 |  |
| Surgery | BCS | 83 | 32.8 | 167 | 32.9 | 0 | 24 | 28.9 | 57 | 34.8 | -0.13 |
|  | Mastectomy | 170 | 67.2 | 341 | 67.1 |  | 59 | 71.1 | 107 | 65.2 |  |
| Axillary surgery | SLNB | 99 | 39.1 | 201 | 39.6 | 0.05 | 24 | 28.9 | 48 | 29.3 | 0.03 |
|  | ALND | 137 | 54.2 | 279 | 54.9 |  | 55 | 66.3 | 107 | 65.2 |  |
|  | No | 17 | 6.7 | 28 | 5.5 |  | 4 | 4.8 | 9 | 5.5 |  |
| ER | Positive | 0 | 0.0 | 0 | 0.0 |  | 78 | 94.0 | 158 | 96.3 |  |
|  | Negative | 253 | 100.0 | 508 | 100.0 |  | 5 | 6.0 | 6 | 3.7 |  |
| PgR | Positive | 0 | 0.0 | 0 | 0.0 |  | 31 | 37.3 | 135 | 82.3 |  |
|  | Negative | 253 | 100.0 | 508 | 100.0 |  | 51 | 61.4 | 29 | 17.7 |  |
|  | Unknown | 0 | 0.0 | 0 | 0.0 |  | 1 | 1.2 | 0 | 0.0 |  |
| HER2 | Positive | 0 | 0.0 | 0 | 0.0 |  | 0 | 0.0 | 0 | 0.0 |  |
|  | Negative | 253 | 100.0 | 508 | 100.0 |  | 83 | 100.0 | 164 | 100.0 |  |
| Neoadjuvant chemotherapy | Yes | 58 | 22.9 | 123 | 24.2 | -0.03 | 22 | 26.5 | 31 | 18.9 | 0.18 |
|  | No | 195 | 77.1 | 385 | 75.8 |  | 61 | 73.5 | 133 | 81.1 |  |
| Type | Anthracyclines | 11 | 19.0 | 21 | 17.1 |  | 3 | 13.6 | 7 | 22.6 |  |
|  | Taxane | 7 | 12.0 | 15 | 12.2 |  | 3 | 13.6 | 4 | 12.9 |  |
|  | Anthracyclines + taxane | 38 | 65.5 | 86 | 69.9 |  | 16 | 72.7 | 20 | 64.5 |  |
|  | Others | 2 | 3.4 | 1 | 0.8 |  | 0 | 0.0 | 0 | 0.0 |  |
| Neoadjuvant molecular targeted therapy | Yes | 3 | 1.2 | 3 | 0.6 | 0.06 | 0 | 0.0 | 0 | 0.0 | 0 |
|  | No | 250 | 98.8 | 505 | 99.4 |  | 83 | 100.0 | 164 | 100.0 |  |
| Neoadjuvant endocrine therapy | Yes | 3 | 1.2 | 3 | 0.6 | 0.06 | 1 | 1.2 | 2 | 1.2 | 0 |
|  | No | 250 | 98.8 | 505 | 99.4 |  | 82 | 98.8 | 162 | 98.8 |  |
| Adjuvant chemotherapy | Yes | 156 | 61.7 | 322 | 63.4 | -0.04 | 44 | 53.0 | 92 | 56.1 | -0.06 |
|  | No | 97 | 38.3 | 186 | 36.6 |  | 39 | 47.0 | 72 | 43.9 |  |
| Type | CMF | 4 | 2.6 | 10 | 3.1 |  | 0 | 0.0 | 1 | 1.1 |  |
|  | Anthracyclines | 42 | 26.9 | 83 | 25.8 |  | 12 | 27.3 | 27 | 29.3 |  |
|  | Taxane | 37 | 23.7 | 63 | 19.6 |  | 9 | 20.5 | 24 | 26.1 |  |
|  | Anthracyclines + taxane | 44 | 28.2 | 109 | 33.9 |  | 18 | 40.9 | 33 | 35.9 |  |
|  | Others | 29 | 18.6 | 57 | 17.7 |  | 5 | 11.4 | 7 | 7.6 |  |
| Adjuvant molecular targeted therapy | Yes | 2 | 0.8 | 2 | 0.4 | 0.05 | 2 | 2.4 | 1 | 0.6 | 0.15 |
|  | No | 251 | 99.2 | 506 | 99.6 |  | 81 | 97.6 | 163 | 99.4 |  |
| Adjuvant endocrine therapy | Yes | 5 | 2.0 | 14 | 2.8 | -0.05 | 48 | 57.8 | 99 | 60.4 | -0.05 |
|  | No | 248 | 98.0 | 494 | 97.2 |  | 35 | 42.2 | 65 | 39.6 |  |
| Adjuvant radiotherapy | Yes | 86 | 34.0 | 174 | 34.3 | -0.01 | 27 | 32.5 | 57 | 34.8 | -0.05 |
|  | No | 167 | 66.0 | 334 | 65.7 |  | 56 | 67.5 | 107 | 65.2 |  |
| Irradiated site | Breast | 56 | 65.1 | 113 | 64.9 |  | 16 | 59.3 | 35 | 61.4 |  |
|  | Chest wall | 9 | 10.5 | 13 | 7.5 |  | 2 | 7.4 | 5 | 8.8 |  |
|  | Breast + RNI | 2 | 2.3 | 5 | 2.9 |  | 2 | 7.4 | 2 | 3.5 |  |
|  | Chest wall + RNI | 16 | 18.6 | 33 | 19.0 |  | 6 | 22.2 | 9 | 15.8 |  |
|  | Others | 3 | 3.5 | 10 | 5.7 |  | 1 | 3.7 | 6 | 10.5 |  |

SCC: squamous cell carcinoma; IDC: invasive ductal carcinoma; SMD: standardized mean difference; BCS: breast-conserving surgery; SLNB: sentinel lymph node biopsy; ALND: axillary lymph node dissection; ER: estrogen receptor; PgR: progesterone receptor; HER2: human epidermal growth factor receptor 2; CMF: cyclophosphamide endoxan fluorouracil; RNI: regional nodal irradiation

Figure 1a


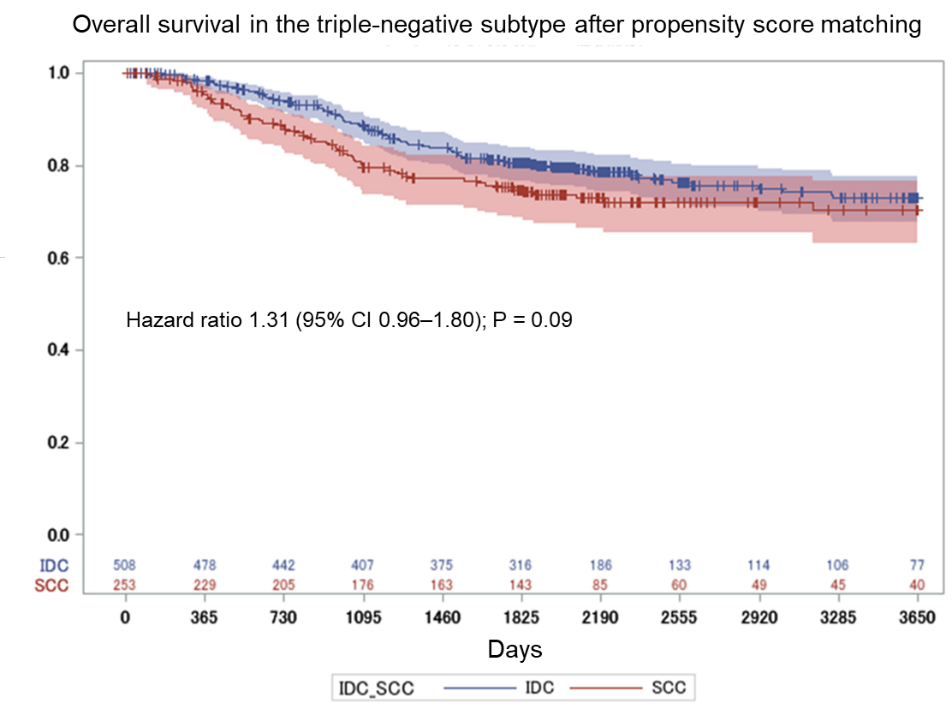


Figure 1b


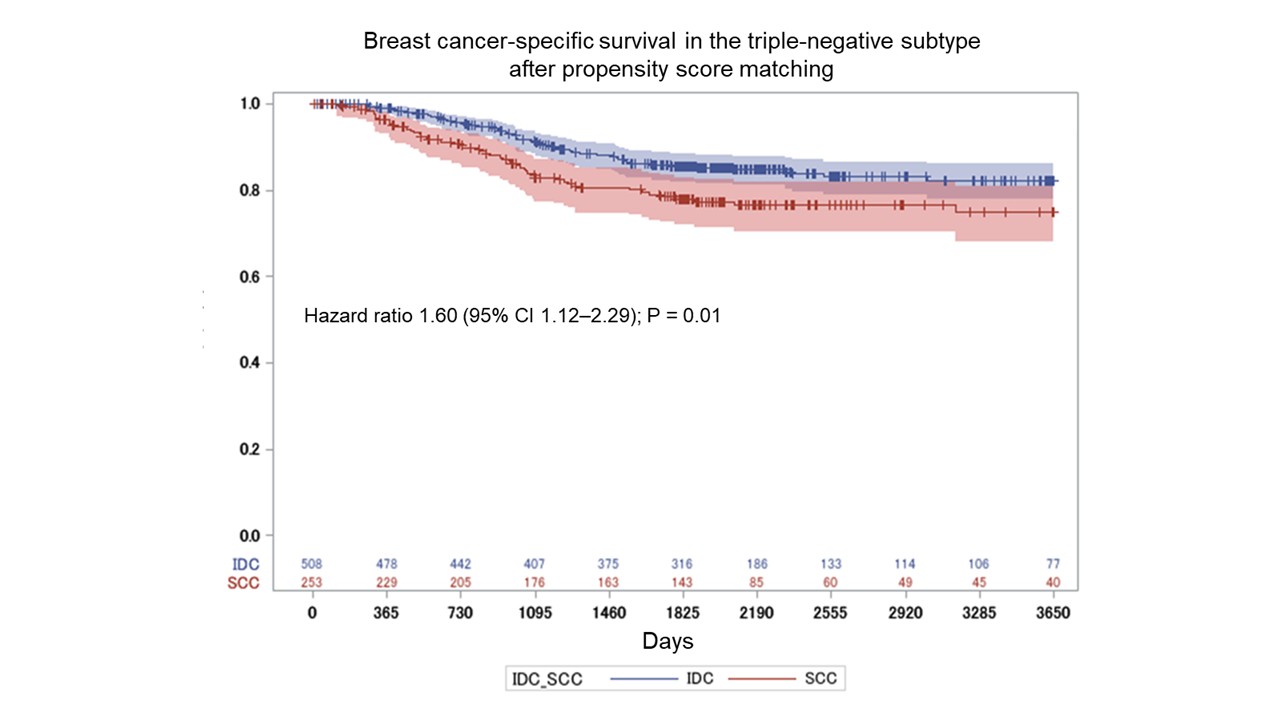


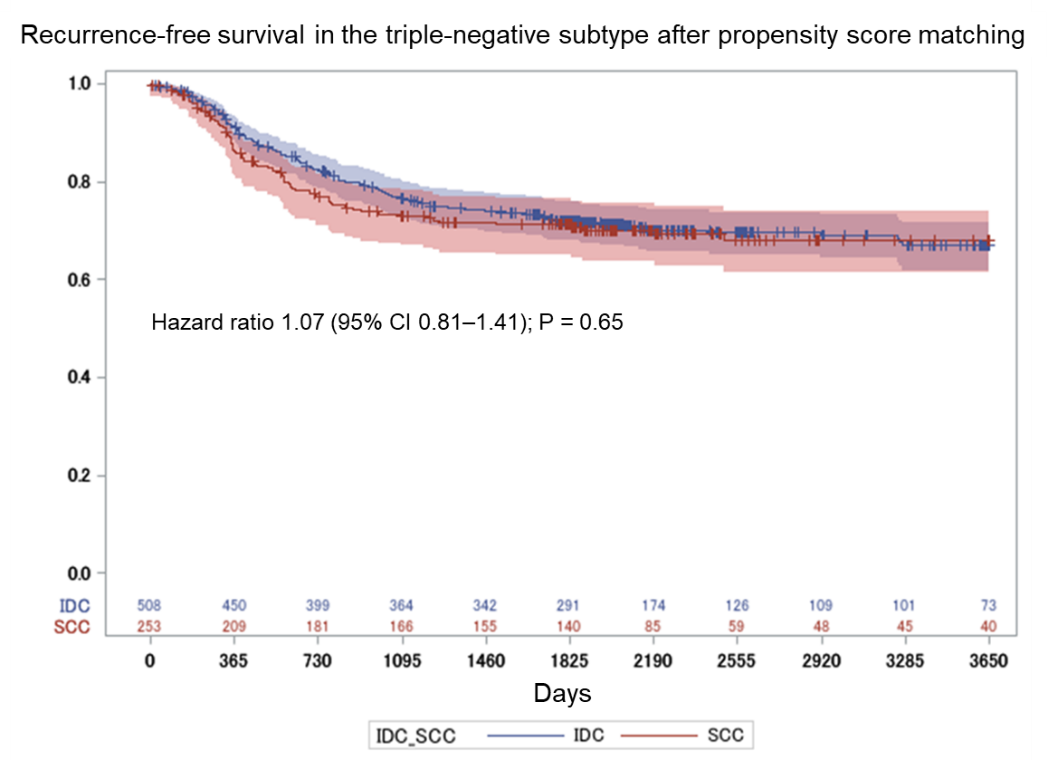
Figure 1c

Figure 1d


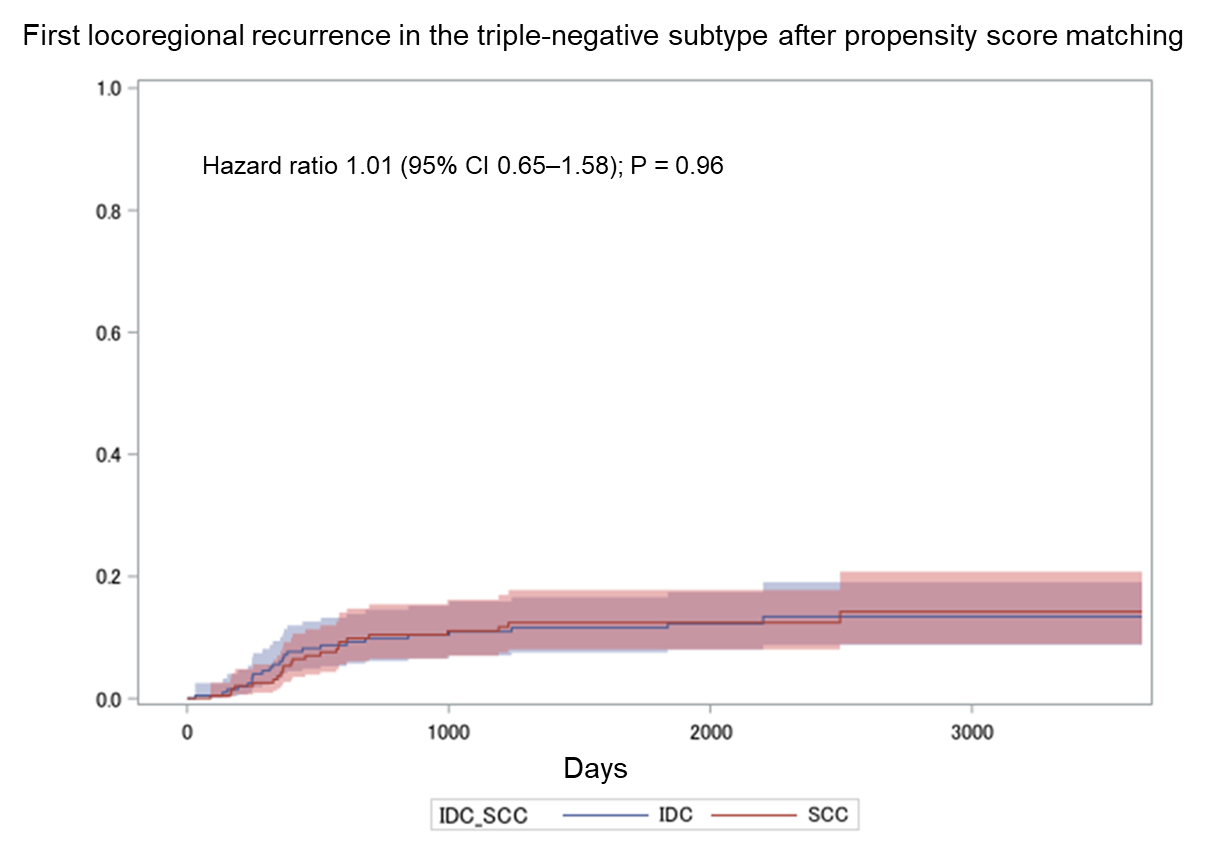


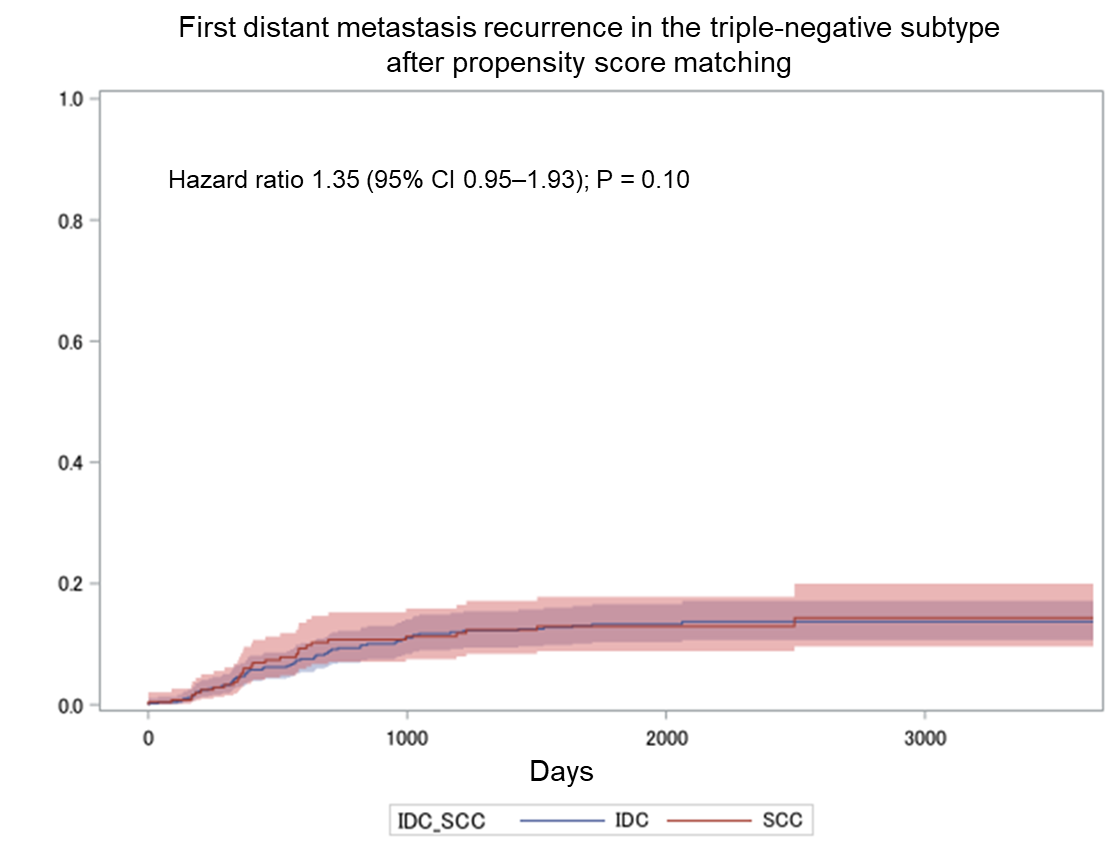
Figure 1e

Supplementary Figure 1

Survival and recurrence outcomes in the triple‑negative subtype after propensity score matching between SCC and IDC patients: (a) overall survival, (b) breast cancer-specific survival, (c) recurrence‑free survival, (d) cumulative incidence of first locoregional recurrence, and (e) cumulative incidence of first distant metastasis

SCC: squamous cell carcinoma; IDC: invasive ductal carcinoma


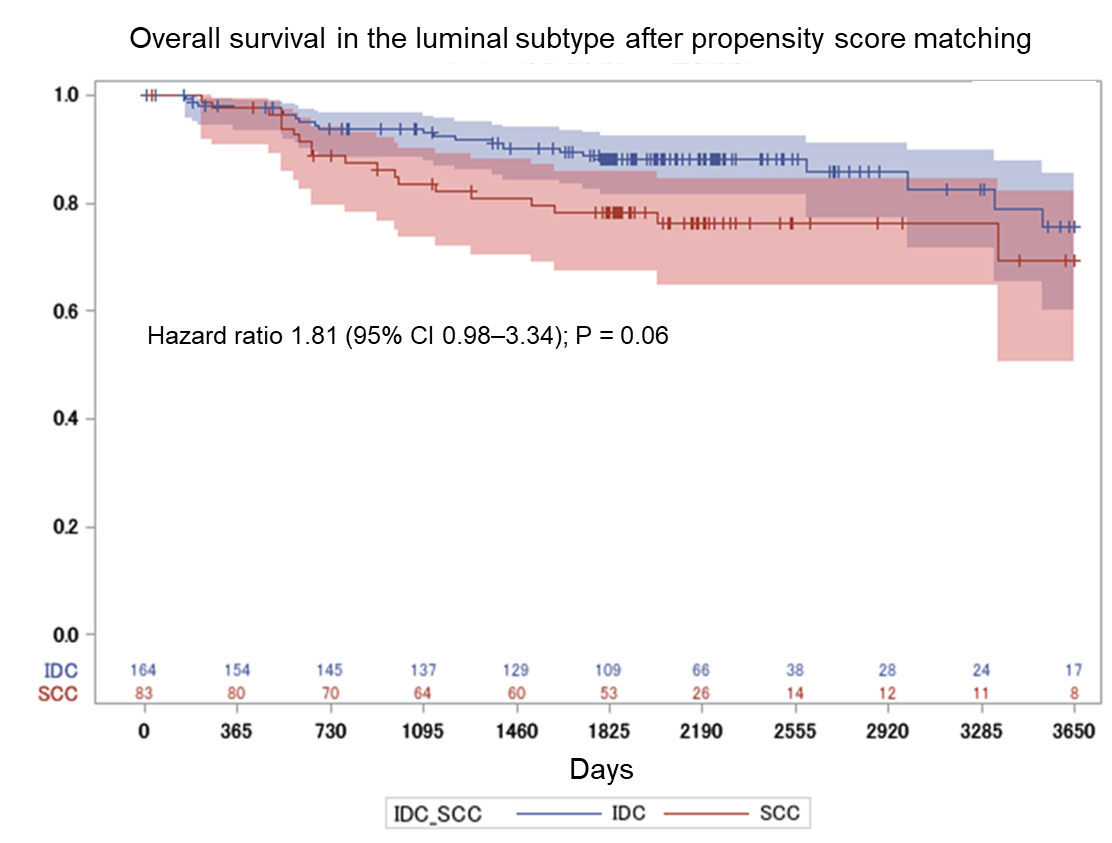
Figure 2a

Figure 2b


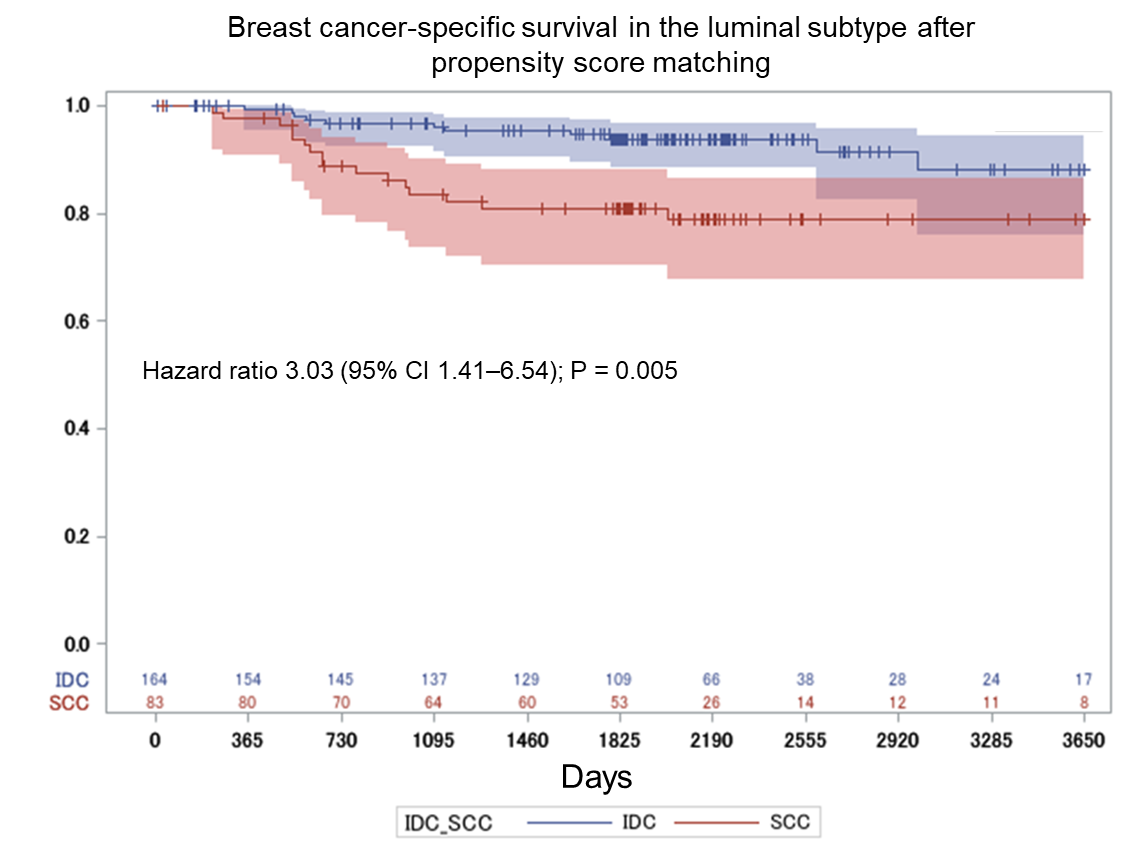


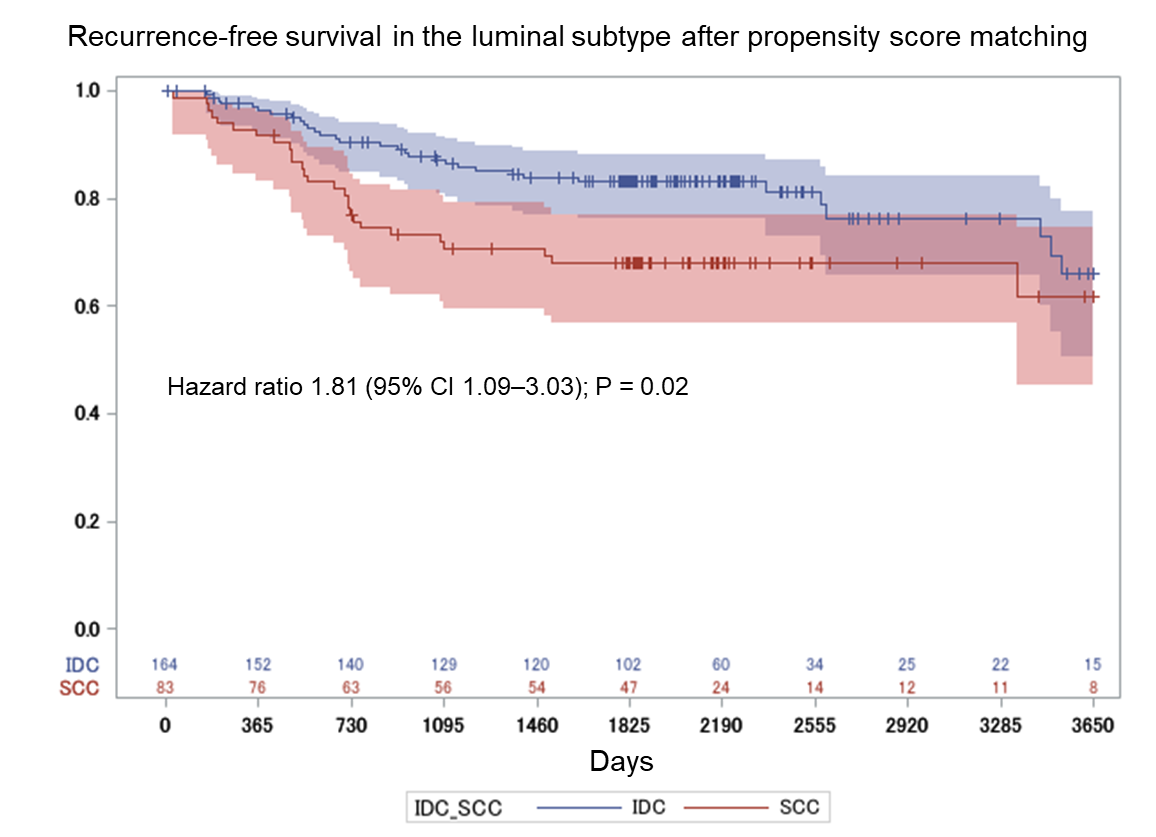
Figure 2c

Figure 2d


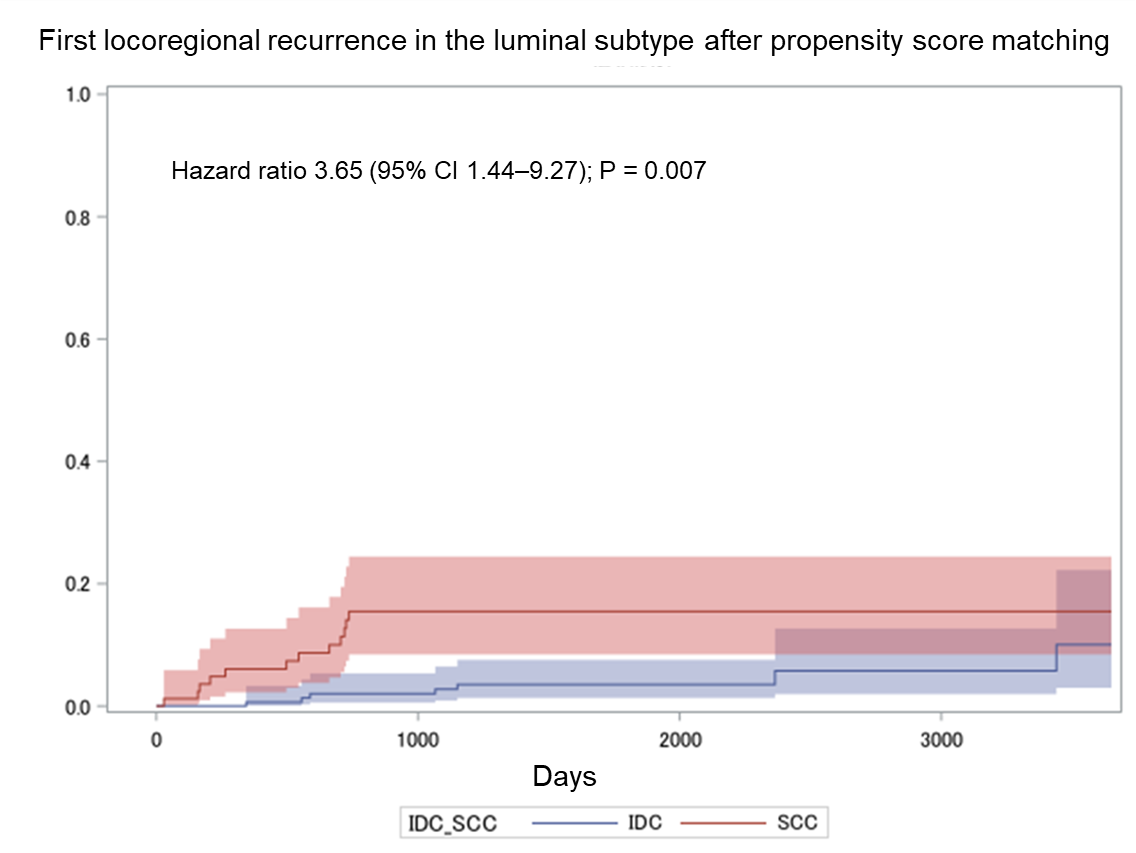


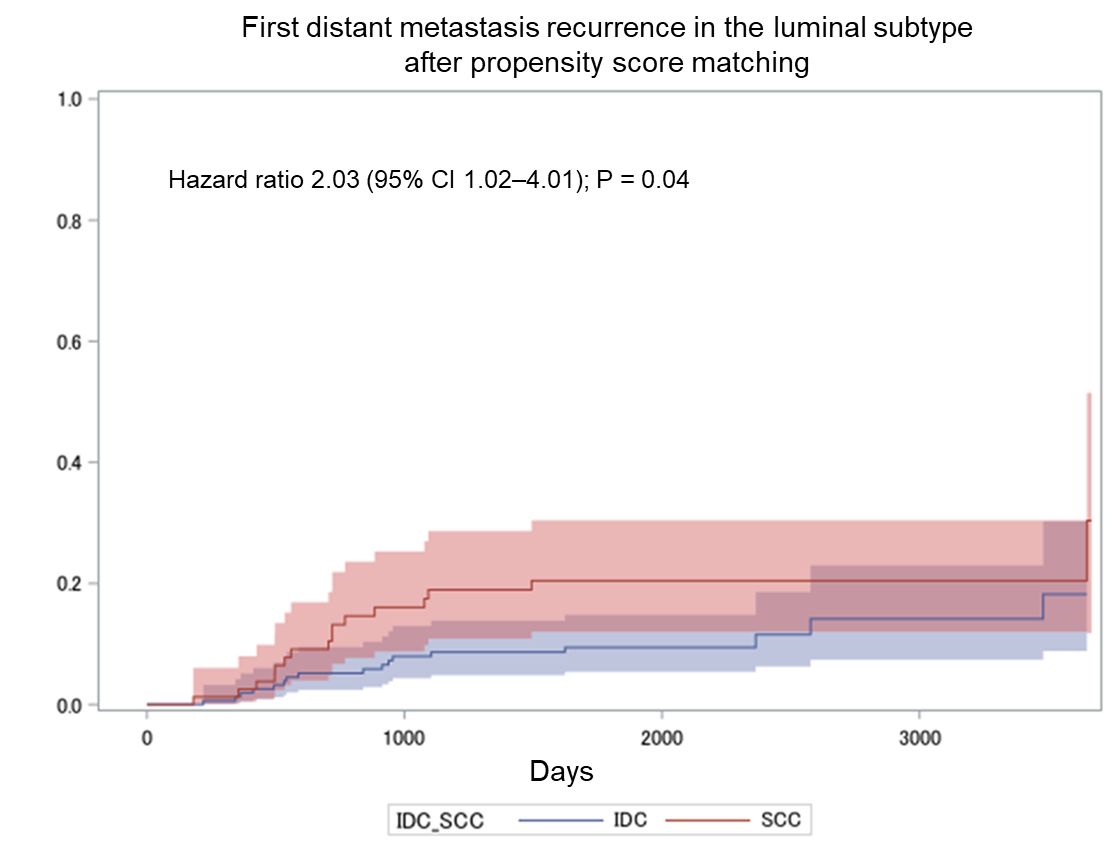
Figure 2e

Supplementary Figure 2

Survival and recurrence outcomes in the luminal subtype after propensity score matching between SCC and IDC patients: (a) overall survival, (b) breast cancer-specific survival, (c) recurrence‑free survival, (d) cumulative incidence of first locoregional recurrence, and (e) cumulative incidence of first distant metastasis

SCC: squamous cell carcinoma; IDC: invasive ductal carcinoma
